# Genomic Predictive Biomarkers in Breast Cancer: The *Haves* and *Have Nots*

**DOI:** 10.3390/ijms26157300

**Published:** 2025-07-28

**Authors:** Kate Beecher, Tivya Kulasegaran, Sunil R. Lakhani, Amy E. McCart Reed

**Affiliations:** 1 UQ Centre for Clinical Research, Faculty of Health, Medicine and Behavioural Sciences, The University of Queensland, Brisbane 4029, Australias.lakhani@uq.edu.au (S.R.L.); 2 Royal Brisbane and Women’s Hospital, Herston, Brisbane 4029, Australia; 3 Pathology Queensland, The Royal Brisbane and Women’s Hospital, Brisbane 4029, Australia; 4 Sullivan Nicolaides Pathology, Brisbane 4006, Australia

**Keywords:** genomic, biomarkers, breast cancer

## Abstract

Precision oncology, also known as personalized oncology or precision medicine, is the tailoring of cancer treatment to individual patients based on the specific genetic, molecular, and other unique characteristics of their tumor. The goal of precision oncology is to optimize the effectiveness of cancer treatment while minimizing toxicities and improving patient outcomes. Precision oncology recognizes that cancer is a highly heterogeneous disease and that each patient’s tumor has a distinct genetic diversity. Precision medicine individualizes therapy by using information from a patient’s tumor in the context of clinical history to determine optimal therapeutic approaches and increasing numbers of drugs target specific tumor alterations. Several targeted therapies with approved companion diagnostics are commercially available, the *haves* of precision oncology, where predictive biomarkers guide clinical decision-making and improve outcomes. However, many therapies still lack clear biomarkers, the *have nots*, posing a challenge to fully realizing the promise of precision oncology. Herein, we describe the current state of the art for breast cancer precision oncology and highlight the therapeutic agents that require a more robust biomarker.

## 1. Introduction

Breast cancer (BC) is a complex and heterogeneous disease. According to the most recent WHO guidelines [1] there are ~20 different histological subtypes of BC, which display differences in morphology and growth pattern. Accounting for ~80% of all cases is Invasive Carcinoma of No Special Type (IC-NST; previously called Invasive Ductal Carcinoma (IDC)) [2]. The remaining ~20% are classified as ‘special’ histological types as they have characteristic features. The most common special type is Invasive Lobular Carcinoma (ILC), accounting for up to 15% of cases [2,3]. The clinical classification of BC centers on three clinical biomarkers: estrogen receptor (ER), progesterone receptor (PR), and human epidermal growth factor receptor 2 (HER2). The absence of which determines a ‘triple negative’ status (triple negative breast cancer, TNBC). Targeted therapies for BC can be administered in the adjuvant or neo-adjuvant setting as well as in the metastatic setting.

Over time, BC survivorship has increased significantly, and now we need to consider the quality of the extra life years being afforded to BC survivors. Side effects from therapy can be acute or chronic, with some effects persisting long after treatment cessation. In many cases, effects can be relatively well managed pharmaceutically (e.g., diarrhea, skin rashes, etc.); however, others will suffer more severe adverse effects and require in-patient management. The targeting of drugs to specific mutations or molecules can reduce ‘off-target’ effects in many instances, however for some, this generates additional consequences. For example, alpelisib (see below) frequently causes hyperglycaemia [4].

Genomic biomarkers are increasingly recognized as a critical complement to the gold standard immunohistochemistry (IHC), offering greater sensitivity and specificity, reduced subjectivity, and quantitative and reproducible results. Unlike IHC, genomic assays provide objective insights into specific molecular alterations, enabling more precise disease characterization and the identification of actionable targets. For example, *PIK3CA* mutations represent a genomic biomarker *have*, guiding the use of PI3K inhibitors in hormone receptor-positive (HR+) BC. In contrast, other therapies remain *have nots*, lacking robust (genomic) predictors to inform their use. This added molecular resolution supports more tailored and reliable treatment decisions, reinforcing the role of genomics in advancing precision oncology. Broadly, biomarkers can be considered either prognostic (indicative of an association with survival outcome), predictive (indicative of a likely response to a specific therapy) or both.

**Figure 1:**
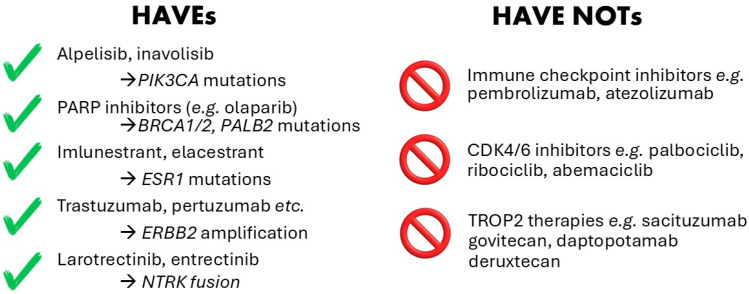
The *haves* and *have nots* of genomic biomarkers in breast cancer.

A standard approach to genomic characterization in both early and advanced BC is lacking, both in terms of method and timing. Different centers apply gene panel sequencing (MSK Impact [5]), exome sequencing [6] or whole genome sequencing [7] either at the time of diagnosis, or at failure of first-line treatment. While some therapies have reliable genomic biomarkers which have now moved from being exclusively used in the metastatic setting towards routine implementation in early BC (e.g., PARP inhibitors), for several therapies no biomarkers, genomic or otherwise, have yet been identified. The evidence for a genomic biomarker’s response to a specific therapy is ranked by international guidelines such as the European Society of Medical Oncology (ESMO) Scale for Clinical Actionability of molecular Targets (ESCAT; [8] or OncoKB [9,10]. The guidelines are informed by clinical trials and Food and Drug Authority (FDA) approvals, as well as emerging research. As of June 2025, OncoKB, the Memorial Sloan Kettering Precision Oncology database, notes 55 genes with FDA-approved targeted therapies with Level 1 evidence, with 152 drugs. For BC, 57 drugs and 30 actionable genes are recorded (Table 1; survival impacts are included in Table 2). As many of the latest generation therapies are accompanied by unpleasant and occasionally debilitating side effects, judicious prescription of the drugs is needed. Together with high drug costs, clinicians, and healthcare systems more broadly, seek conclusive biomarkers indicative of efficacy. Below, we consider the *haves*, drugs with reliable genomic predictive biomarkers, and the *have nots,* those still in need of improved biomarker options (Figure 1).

## 2. Genomic Biomarkers in Breast Cancer: *The Haves*

### 2.1. Homologous Recombination Deficiency (HRD) and PARP Inhibitors

Homologous recombination deficiency (HRD) is marked by genomic instability resulting from dysfunction in BRCA1/2 or other homologous recombination and repair (HRR) proteins. Breast cancer patients exhibiting an HRD phenotype, with or without detected mutations in HRR genes, have shown benefit from Poly (ADP-ribose) polymerase (PARP) inhibitor (PARPi) treatment [30]. PARP inhibitors block the repair of single-strand DNA breaks by inhibiting PARP enzymes, leading to the accumulation of DNA damage [30]. In cancer cells with defective homologous recombination (like *BRCA1/2* mutations), this results in double-strand breaks that cannot be repaired, causing cell death [30].

The current biomarker predictive of PARPi sensitivity is germline *BRCA1/2* (*gBRAC1/2*) and *PALB2* mutation [31], with emerging evidence for somatic (*sBRCA1/2*) mutations. In the phase III OlympiA trial with 1836 early high-risk HER2- BC patients with confirmed *BRCA1* or *BRCA2* germline mutations, second-line olaparib treatment led to significantly longer disease-free survival (DFS) and overall survival (OS) [11,12]. Olaparib benefit was consistent across all groups, with fewer malignancies in the treatment group [32]. In the phase III EMBRACA trial, 431 advanced BC patients with *gBRCA1/2* mutation showed talazoparib provided a significant benefit over standard chemotherapy with respect to progression-free survival (PFS) in TNBC and ER+ disease [13]. A meta-analysis has shown no significant difference in response between *gBRCA1/2* and *sBRCA1/2* alterations and PARPi [33].

### 2.2. PI3K/AKT/mTOR Pathway

The PI3K/AKT/mTOR (phosphatidylinositol 3-kinase/protein kinase B/mammalian Target of Rapamycin) signaling pathway is highly conserved and plays a critical role in cell growth and cell cycle progression. Activation of PI3K leads to phosphorylation of AKT, which in turn activates mTOR, a central regulator of protein synthesis and cell cycle progression [34]. When dysregulated, the pathway can act as an oncogenic driver of proliferation in cancer, while also being implicated in a range of diseases such as diabetes, cardiovascular disease and neurodegenerative diseases such as Alzheimer’s Disease. Pathway activation is reported in 50% of all cancers, making it the most commonly activated signaling pathway in cancer, and an appealing therapeutic target [34] despite the high risk of off-target effects at therapeutic doses.

PI3K catalytic subunit (*PIK3CA*) mutations are detected in approximately 30% of BC, they are notably more common in ER+ and HER2+ diseases, with prevalence rates of 39% and 37%, respectively [35]. *PIK3CA* mutations predict response to a targeted therapy (alpelisib) but also to likely resistance to chemotherapy. The Phase III SOLAR-1 trial showed benefit of alpelisib plus fulvestrant in those *PIK3CA*-mutated HR+ HER2- advanced BC patients who had progressed on or after prior aromatase inhibitor therapy mutation [14]. Alpelisib can also bind to wild-type *PIK3CA* (PI3K protein), which results in pronounced off-target effects, such as hyperglycaemia [4]; however, inavolisib and RLY-2608 (emerging drug heading into Phase III trials) specifically target mutant PI3K. The phase III INAVO120 trial showed that in patients with *PIK3CA*-mutated, HR+ HER2- advanced breast cancers, treatment with inavolisib (plus palbociclib and fulvestrant) led to a significant overall survival benefit, although adverse effects were more frequent with inavolisib [15]. The Phase III SANDPIPER trial also demonstrated that, in patients *PIK3CA*-mutated, HR+ HER2- ABC, treatment with taselisib plus fulvestrant resulted in a significantly longer PFS than fulvestrant alone [16]. Similarly, in the neoadjuvant LORELEI study, the combination of taselisib plus letrozole significantly improved objective response rate (ORR) compared to placebo plus letrozole in the ER+, HER2- patients and *PIK3CA*-mutant population achieved greater benefit [17].

Targeting AKT is also an attractive treatment option. The serine/threonine kinase AKT (otherwise known as protein kinase B) is a key element in the PI3K/AKT/mTOR pathway. This pathway is commonly deregulated in HR, HER2 and TNBC subtypes. There are three AKT isoforms (AKT1, AKT2, and AKT3) that are encoded by different genes with AKT1 and AKT2 being ubiquitously distributed [36] and AKT3 being predominantly expressed in neural cells [37]. Targeting AKT is a treatment option for those resistant to conventional treatments with several inhibitors available including capivasertib and ipatasertib. In addition, there are many allosteric inhibitors in development (e.g., ARQ092/miransertib; BAY1125976; MK-2206; TAS-117) and some are in early clinical trials for other tumor types, with only MK-2206 being tested in BC [38,39,40,41,42]. In the phase II FAKTION trial [18,19], an AKT inhibitor combined with endocrine therapy resulted in no benefit observed among patients with *PI3K/PTEN* altered tumors [18,19]. In CAPItello-291, capivasertib in combination with fulvestrant for the treatment of HR+/HER2- advanced BC with one or more *PIK3CA/AKT1/PTEN* alterations significantly improved PFS [20]. The phase III IPATunity130 trial showed that the addition of the ATK inhibitor ipatasertib did not improve efficacy in patients with *PI3K/AKT1/PTEN* mutations [21]. In the PAKT trial, TNBC patients with *PIK3CA/AKT1/PTEN*-altered tumors had an improved median PFS of 9.3 months with capivasertib plus paclitaxel compared to 3.7 months with placebo [43]. Similarly in the LOTUS trial, ipatasertib plus paclitaxel resulted in an enhanced survival benefit in TNBC patients with *PIK3CA/AKT/PTEN* mutational status [44,45]. Overall, AKT inhibitors have shown a lack of correlation between PI3K/AKT pathway alterations and efficacy of AKT inhibitors from HR+ BC patients with limited data available on HER2+ and a positive trend in TNBC patients.

Although the role of the AKT/PI3K/mTOR pathway in driving cellular transformation is well defined, our understanding of the impact of mutation on the biology of the disease and its role in determining therapy response in patients is nascent, particularly in the therapy resistance space. *PIK3CA* mutations predict resistance to a range of chemotherapies. In the neoadjuvant setting, a Phase III trial in HER2+ BC showed *PIK3CA* mutations were associated with poorer outcomes in all treatment groups (NeoALTTO) [46]. Patients with wild-type *PIK3CA* receiving a combination of trastuzumab and lapatinib achieved a pathologic complete response (pCR) rate of 53.1%. However, this rate decreased to 28.6% in patients with tumors carrying *PIK3CA* activating mutations. Indeed, a 13–20% absolute decrease in complete response rates in patients with *PIK3CA*-mutant versus wild-type tumors has been reported [47,48]. The status of *PIK3CA* is crucial for personalized therapeutic strategies in BC.

### 2.3. ESR1

While first-line endocrine therapy provides effective control of early disease in HR+ BC, resistance often emerges over time. A critical driver of this resistance is mutation of the *ESR1* ligand-binding domain, which encodes estrogen receptor alpha (ERα). ERα activation promotes proliferation, making *ESR1* a key driver in HR+ BC. Mutations or overexpression of *ESR1* can lead to endocrine resistance and the occurrence of these mutations typically depends on the duration and context of prior endocrine therapy [49]. *ESR1* mutations are relatively prevalent in metastatic BC, with around 20–40% of patients treated with aromatase inhibitors (AI) exhibiting these mutations [50]; such mutations are thought to account for resistance in 50% of metastatic breast cancer (mBC) patients. Additionally, mechanisms such as *ESR1* loss, amplification, and translocation contribute to the complexity of ER biology [51]. Emerging ER-targeting therapies are proving efficacious even in those patients with acquired *ESR1* mutations. For example, elacestrant demonstrated significant efficacy with an extended PFS, particularly for patients with *ESR1* mutations in the EMERALD trial [22,52]. Lasofoxifene, a novel selective ER modulator, has also been shown to improve PFS and clinical benefit rate in *ESR1*-mutated patients that progressed on an AI plus a CDK4/6 inhibitor (ELAINE 1 trial) [23]. Kingston and colleagues examined genomic factors influencing response and resistance in Cohort A of the plasmaMATCH trial [53] and found that *ESR1* mutations in ctDNA correlated with outcomes. Notably, baseline *ESR1* Y537S mutations were linked to poorer outcomes, while Y537C mutations showed favorable results [53]. In the phase III EMBER-3 trial, patients with ER+ HER2- advanced BC had significantly longer PFS with imlunestrant compared to standard therapy, but only among those with *ESR1* mutations; no benefit was observed in the overall population [15]. Additionally, they discovered *ESR1* F404 mutations, which develop alongside existing activating *ESR1* mutations, served as a resistance mechanism against fulvestrant [53].

Monitoring the emergence and persistence of *ESR1* mutations in blood samples presents a promising non-invasive opportunity for real-time monitoring of disease recurrence, and for informing therapeutic strategy. Currently, two trials, PADA-1 [54] and SERENA-6 [55], targeting ESR mutations in the advanced BC setting and have shown an improvement in progression-free survival (PFS). However, we are still awaiting results on OS and time to second progression event (PFS2), and these strategies are not yet standard practice.

### 2.4. HER2/ERBB2 Mutations

HER (human epidermal growth factor receptor) family proteins, including HER2, regulate cell proliferation by activating downstream signaling pathways like PI3K/AKT/mTOR and MAPK upon ligand binding or receptor dimerization. Amplification or overexpression of *ERBB2*/HER2 leads to uncontrolled cell growth and is a major driver of certain aggressive breast cancers. While amplification of the *ERBB2* gene intrinsically defines the HER2+ class of BC and is a long-established predictive biomarker of response to HER2 targeting therapies such as trastuzumab and pertuzumab, the mutation status of the gene and its binding partner HER3/*ERBB3* are now emerging as potential predictive biomarkers. Mutations in *ERBB2* and *ERBB3* were shown to activate the epidermal growth factor (EGF) pathway nearly a decade ago [56], with in vitro sensitivity to neratinib and resistance to lapatinib. Indeed, these mutations are indicators of poor prognosis in lobular BC [57,58], and are acquired in the metastatic setting in a resistance response toward endocrine therapies [59,60]. The interplay between the ER and EGF pathways has necessitated variations in dosing strategies to counteract resistance, and current approaches consider neratinib with trastuzumab, as well as fulvestrant [61]. Although relatively rare, the mutation status of *ERBB2/3* are likely to move toward higher evidence levels on the OncoKB and ESCAT scales.

Antibody-drug conjugates (ADCs) have significantly changed the therapeutic landscape in BC, with FDA-approved ADCs targeting HER2/3, including trastuzumab deruxtecan (T-DXd) and trastuzumab emtansine (T-DM1) [25,62]. Findings from clinical trials such as DESTINY-Breast04 [24] have expanded the use of HER2-directed therapies to patients with HER2-low and even HER2-ultralow expression, broadening the eligible patient population for such drugs. The biological and clinical relevance of low/ultralow HER2 expression remains unclear, raising questions about whether HER2-low status reflects a distinct disease subtype or simply a spectrum of HER2 expression. It remains to be seen whether HER2 will continue to be an appropriate biomarker with the emergence of HER2 low and ultra-low groups.

### 2.5. NTRK

Genetic biomarkers can be applied across multiple cancer types, e.g., HER2 (*ERBB2*) amplification, and the first genetic biomarker to be used in a cancer agnostic approach was *NTRK* fusions (Neurotrophic tyrosine receptor kinase) [63]. Secretory BC is an example of a rare, special histological subtype of TNBC and comprehensive genome profiling has identified a pathognomonic chromosomal translocation (t(12; 15) (p13; q25)) involving *NTRK* driving the phenotype [64,65]. NTRK targeting (e.g., larotrectinib, entrectinib) has resulted in durable responses [26,63,66].

## 3. Efficacious Therapies Awaiting Improved Biomarkers for Precision Application in Breast Cancer: *The Have Nots*

### 3.1. CDK4/6 Inhibitors

Many HR+ BC patients experience relapse within a decade of diagnosis [67], and the application of targeted therapies like Cyclin-dependent kinases 4 and 6 (CDK4/6) inhibitors (e.g., ribociclib, abemaciclib, and palbociclib) in combination with endocrine therapy (ET, e.g., like tamoxifen and aromatase inhibitors (AIs)), has changed the management of these patients. *CDK4* and *CDK6* and their protein regulator cyclin D1 (encoded by *CCND1*), are direct transcriptional targets of ER signaling and regulate cell-cycle progression. While CDK4/6 inhibitors are generally well-tolerated, the financial impact to both patients and the health service is significant, and more judicious prescription of these therapies is needed. Various potential biomarkers of CDK4/6 activity have been studied across a complex clinical trial landscape, but none have proven to be clinically useful in the context of CDK4/6 inhibitors. An initial analysis of 2242 BC patients who underwent both germline and somatic sequencing revealed *BRCA2* carriers had poorer outcomes when treated with first-line CDK4/6i-ET [68]. In the PALOMA-3 trial, a comprehensive gene expression analysis was conducted; however, despite evaluating 2534 cancer-related genes no biomarkers associated with the efficacy of adding palbociclib to fulvestrant in mBC patients were identified (however, high *CCNE1* mRNA expression was associated with resistance to Palbociclib) [69]. This was further supported by the PALOMA-1 trial and the preoperative-palbociclib (POP) study, both of which found *CCND1* amplification was not predictive of Palbociclib response [70]. Consequently, higher *CCNE1* mRNA levels were linked with poorer prognosis in MONALEESA-2 [71], however the MONALEESA-3 and PALOMA-2 studies demonstrated a consistent benefit of CDK4/6i regardless of the levels of *CCNE1* expression [72,73]. In the neoadjuvant setting, increased *CCNE1* amplification was associated with unfavorable outcomes in the NeoPalAna trial [74] and higher *CCNE1* expression tended to be more associated with resistance although this trend was not statistically significant (neoMonarch) [75]. In summary, although findings are somewhat conflicting and vary across treatment settings, *CCNE1* amplification may have relevance as a genomic marker associated with resistance to CDK4/6 inhibitors, highlighting its potential role in guiding therapeutic decisions rather than serving solely as a prognostic indicator.

The primary mechanism of CDK4/6 inhibitor activity is suppression of Retinoblastoma protein (RB) phosphorylation, enforcing G1 cell cycle arrest, thus inhibiting proliferation. Consequently, an intact and functional RB gene is essential for the antiproliferative efficacy of CDK4/6 inhibitors; both gene and protein have been investigated as potential biomarkers for predicting response. In a retrospective study, it was observed that *RB1* loss was associated with a PFS of 3.6 months, whereas functional *RB1* was associated with a longer PFS of 10.1 months [76]. A pooled baseline ctDNA analysis of 1503 patients in the MONALEESA-2, -3, -7 trials showed patients with wild-type *RB1* tended to have slightly longer PFS when treated with ribociclib than mutant *RB1* [51]. A single-arm phase II study evaluated abemaciclib monotherapy in patients with previously treated, RB1-positive (by IHC using the antibody clone G3-245) metastatic TNBC. The treatment, however, demonstrated limited clinical activity in this population [77]. Additional studies in the neoadjuvant setting have shown that *RB1* mutations are relatively rare, suggesting loss of function, rather than genetic mutation alone, may contribute to primary resistance to CDK4/6 inhibitors [74,75]. Interestingly, the NeoPalAna trial introduced the concept of a *CCNE1/RB1* ratio as a better predictor of sensitivity or resistance to palbociclib compared to *CCNE1* or *RB1* alone [55]. While *RB1* loss and the *CCNE1/RB1* ratio show promise as potential biomarkers for predicting responses to CDK4/6 inhibitors, ongoing research is needed to refine their clinical utility.

Other players involved in the CDK4/6 signaling pathways, such as CCND1, CCNE1/2, CDK2/6 and CDKN2A have failed to demonstrate a significant relationship between their baseline gene expression levels and the benefit derived from adding palbociclib to letrozole treatment [72]. This is surprising considering CDKN2A’s compelling biological evidence for biomarker status (Level 4 evidence, OncoKB). However, discovery analyses have indicated that low E2F transcriptional activity is associated with relatively improved efficacy of Palbociclib [69]. In summary, the search for robust genomic biomarkers that can inform the use of CDK4/6 inhibitors in BC remains ongoing.

### 3.2. Immune Checkpoint Inhibitors and Tumor Mutation Burden

Programmed cell death 1 (PD-1)/programmed cell death ligand 1 (PD-L1) checkpoint inhibitors (e.g., pembrolizumab, atezolizumab) have been approved for metastatic TNBC patients positive for PD-L1 expression. PD-1 is an immune checkpoint receptor that limits T cell effector function within tissues and expression in BC is associated with high-risk clinicopathological features [78]. Immunohistochemistry staining for PD-L1 is employed; however, neither the antibodies nor the scoring assessments are standardized, and additional biomarkers are still needed to optimize PD-1/PD-L1 targeting therapeutics in BC [79,80,81].

Tumor mutational burden (TMB), the total number of mutations present in the DNA of tumor cells, is a predictive biomarker for response to immune checkpoint inhibitors. TMB is measured as mutations per megabase (mut/Mb) of DNA. Tumors with high TMB have increased immunogenicity through the generation of neoantigens which can trigger an immune response. As such, high TMB can be considered a prognostic biomarker when treated with immune checkpoint inhibitors; however, broadly speaking, TMB is not a prognostic biomarker in BC [82]. The FDA approved pembrolizumab for use when solid tumors have a TMB of >10 mut/Mb following the KEYNOTE-158 trial [28], however few breast cancers meet this mutation burden. In the phase III KEYNOTE-522 trial evaluating pembrolizumab combined with chemotherapy in patients with high-risk early-stage TNBC, higher tumor mutational burden was associated with improved event-free survival (EFS) [83]. In a retrospective analysis of 11 advanced TNBC cases from the KEYNOTE-012 and -086 trials, high TMB was associated with better response to pembrolizumab [84]. However, the standard 10 mut/Mb cut-off may not fully capture subtype-specific complexity and is not a clinically meaningful cut-off for many BC, where the average TMB is 2.63 mut/Mb [85]. Importantly, in high TMB tumors, more than 60% show APOBEC defects [86], the presence of which correlates with poor response to endocrine therapy with CDK4/6i [87,88].

High mutational burden can drive resistance to kinase inhibitors by promoting genetic heterogeneity and the emergence of resistant clones with secondary mutations in the target kinase or bypass pathways. As shown in Offin et al. [89], increased mutation rates in lung cancer models led to reduced efficacy of EGFR inhibitors due to the rapid development of resistance mechanisms.

### 3.3. TROP2

Human trophoblast cell surface antigen 2 (TROP2), also termed epithelial glycoprotein 1 (EGP1), a transmembrane calcium signal transducer, plays a role in cancer-cell growth [90,91]. TROP2 is highly expressed in BC and is associated with worse survival [92]. Sacituzumab Govitecan (SG; Trodelvy or IMMU-132) is an ADC approved for use in pre-treated BC patients: an anti-TROP2 mAb delivers the cytotoxic SN-38 directly to the TROP2-expressing tumor cells. Numerous clinical trials have demonstrated the effectiveness of Sacituzumab Govitecan as an untargeted therapy; side effects include neutropenia and diarrhea. Relative benefit of SG was assessed in a Q-TWiST analysis of the TROPiCS-02 trial, and demonstrated a positive benefit risk profile in the pretreated HR+/HER2- mBC setting, when considering ≥ grade 3 adverse effects [29]. An exploratory biomarker analysis from the ASCENT trial found SG-treated patients with high/medium TROP2 expression had longer overall survival and better objective response rate [93]. We await data from the trials investigating anti-TROP2 ADCs in the early BC setting.

## 4. Horizon Scanning for Genomic Biomarkers in BC

Emerging genomic biomarkers in BC research hold promise for improving diagnostic and treatment strategies. NF1, also known as Neurofibromin 1, is being investigated for its role in BC development and progression [84]. Clinical trials, such as NCT05554354, are currently underway to explore the use of binimetinib to target *NF1* mutations in ER+ BC patients. GATA3, a transcription factor involved in breast tissue development, has emerged as a biomarker of interest with the potential for targeting. A subset of *GATA3* mutations occurring in the absence of *TP53* mutation, and with co-amplification of *MDM2* may benefit from MDM2 inhibitors, with a slew of drugs in early trials across cancer types [85,86].

Ongoing research efforts and clinical trials (summarized in Table 2) aimed at identifying genomic biomarkers are critical to the robust implementation of precision oncology across all BC subtypes. Bridging the gap between the *haves* (therapies with validated biomarkers) and the *have nots* (those lacking predictive tools) will not only improve patient outcomes but also deliver significant health economic benefits by minimizing unnecessary toxicity and ensuring therapies are prescribed where there is a high likelihood of benefit.

## Figures and Tables

**Table 1:** Current actionable genomic biomarkers in breast cancer with Level 1 evidence.

Gene/Biomarker	Alteration Type	Cancer Type	Drugs
*AKT1*	Oncogenic Mutations	Breast Cancer	Capivasertib + Fulvestrant
*BRAF*	V600E	All Solid Tumors (excl CRC)	Dabrafenib + Trametinib
*ERBB2*	Amplification	Breast Cancer	Ado-Trastuzumab Emtansine; Lapatinib + Capecitabine, Lapatinib + Letrozole; Margetuximab + Chemotherapy; Neratinib, Neratinib + Capecitabine; Trastuzumab + Pertuzumab + Chemotherapy; Trastuzumab + Tucatinib + Capecitabine; Trastuzumab Deruxtecan; Trastuzumab, Trastuzumab + Chemotherapy
*ESR1*	D538, E380, L469V, L536, S463P, Y537	Breast Cancer	Elacestrant, Camizestrant
*NTRK1*	Fusions	All Solid Tumors	Entrectinib; Larotrectinib
*NTRK2*	Fusions	All Solid Tumors	Entrectinib; Larotrectinib
*NTRK3*	Fusions	All Solid Tumors	Entrectinib; Larotrectinib
*PIK3CA*	H1047R, C420R, E542K, E545A, E545D, E545G, E545K, H1047L, H1047Y, Q546E, Q546R; etc.	Breast Cancer	Alpelisib + Fulvestrant; Capivasertib + Fulvestrant
*PTEN*	Oncogenic Mutations	Breast Cancer	Capivasertib + Fulvestrant
*RET*	Fusions	All Solid Tumors (excl TC)	Selpercatinib
MSI	MSI High	All Solid Tumors	Pembrolizumab
TMB	>10 mut/Mb	All Solid Tumors	Pembrolizumab

CRC, colorectal cancer; TC, thyroid cancer.

**Table 2:** Personalized therapies and patient outcomes in breast cancer.

Drug Class	Genomic Biomarker (Yes or ?)	Drug	Trial	REF	Survival (Treatment vs. PC/SOC)
PARPi	Y	olaparib	OlympiA	[11]	3 yrs DFS (85.9% vs. 77.1%); DDFS (87.5% vs. 80.4%)
				[12]	4 yrs OS (89.8% vs. 86.4%); DFS (82.7% vs. 75.4%); DDFS (86.5% vs. 79.1%)
		talozoparib	EMBRACE	[13]	mDFS (8.6 vs. 5.6 months); ORR (62.6% vs. 27.2%)
PIK3CAi	Y	alpelisib	SOLAR-1	[14]	mOS (39.3 vs. 31.4 months)
		inavolisib	INAVO120	[15]	mOS (34.0 vs. 27.0 months); ORR (62.7% vs. 28.0%)
		taselisib	SANDPIPER	[16]	PFS (7.4 vs. 5.4 months)
			LORELEI	[17]	*PIK3CA* mut subset: ORR 38% vs. 56%
AKTi	Y	capiversatib	FAKTION	[18,19]	In pathway-altered vs. non altered subgroup: mPFS (12.8 vs. 4.6 months); mOS (38.9 vs. 20.0 months)
			CAPItello-291	[20]	PFS (13.0 vs. 12.7 months)
		ipatasertib	IPATunity130	[21]	mPFS 9.3 months; ORR 47%
ET	Y	elacestrant	EMERALD	[22]	mPFS (8.6 vs. 1.9 months); *ESR1* mut (9.0 vs. 1.9 months)
		lasofoxifene	ELAINE 1	[23]	mPFS (24.2 vs. 16.2 weeks); ORR (13.2% vs. 2.9%)
HER ADC	Y	TDXd	DESTINY-Breast04	[24]	mPFS (9.9 vs. 5.1 months); OS (23.4 vs. 16.8 months)
		T-DM1	KATHERINE	[25]	Estimated DFS (88.3% vs. 77.0%)
NTRK fusion	Y	entrectinib	STARTRK-2	[26]	ORR 83% (n = 6)
CDK4/6i	?	palbociclib	e.g., MONALEESA, MONARCH, PALOMA, NATALEE, etc. mBC setting	[27]	Systematic analysis comparing all 3 drugs, mPFS: palbociclib 23.4–31.0 months; ribociclib 19.8–44.0 months; abemaciclib 14.0–39.5 months
		ribociclib			
		abemaciclib			
TMB/ICI	?	pembrolizimab	KEYNOTE 158	[28]	ORR 34.3%, mPFS 4.1 months; mOS 23.5 months.
TROP2	?	sacituzumab govitecan	TROPiCS-02	[29]	OS (14.4 vs. 11.2 months; survival benefit consistent across Trop-2 expression-level subgroups); ORR (21% vs. 14%)

Distant disease-free survival, DDFS; disease-free survival, DFS; metastatic breast cancer, mBC; median disease-free survival, mDFS; median overall survival, mOS; median progression free survival, mPFS; objective response rate, ORR; overall survival, OS; physician’s choice, PC; standard of care, SOC.
